# Community assembly mechanisms and succession processes significantly differ among treatments during the restoration of *Stipa grandis* – *Leymus chinensis* communities

**DOI:** 10.1038/s41598-019-52734-0

**Published:** 2019-11-08

**Authors:** Ke Dong, Guang Hao, Nan Yang, Jian-li Zhang, Xin-feng Ding, Hui-qin Ren, Jun-fang Shen, Jin-long Wang, Lin Jiang, Nian-xi Zhao, Yu-bao Gao

**Affiliations:** 1 Department of Plant Biology and Ecology, College of Life Science, Nankai University, Tianjin, 300071 P.R. China; 2 College of Agronomy & Resources and Environment, Tianjin Agricultural University, Tianjin, 300384 P.R. China; 3 School of Biology, Georgia Institute of Technology, Atlanta, GA 30332 USA

**Keywords:** Biodiversity, Grassland ecology, Grassland ecology

## Abstract

Understanding community assembly mechanisms is helpful to predict community dynamics. To explore which community assembly mechanism(s) drive(s) the grassland restoration in semi-arid region, we investigated the relationships between plant trait and species relative abundance (SRA), and estimated community functional diversity indices for each community under different treatments (enclosure, grazing and mowing treatment) in a restoration region of *Stipa grandis* – *Leymus chinensis* communities in the northern China from 2010 to 2012. There was a high fraction of significant relationships between trait value and SRA, suggesting that niche theory structured the grassland restoration in this region. The functional richness was higher and the functional divergence was lower in the enclosure community than that in the grazing or mowing community, and significantly positive plant height - SRA relationship was found in the enclosure community. These findings demonstrated that limiting similarity based on niche theory was more important in structuring the enclosure community and that environmental filtering based on niche theory played a more important role in driving the grazing or mowing community. Only the factor of year significantly affected the functional evenness (FEve), and the lowest FEve in 2011 implied that the relatively lower precipitation could enhance the effect of limiting similarity on community assembly in the semi-arid grassland.

## Introduction

The restoration of degraded communities is one of the most important challenges in applied ecology^1^. Understanding community assembly mechanisms during the communities’ restoration is helpful for us to know about and forecast the community dynamics during the secondary succession^2,3^. The community assembly mechanisms fall into niche theory (including environmental filtering and limiting similarity) and neutral theory (including stochastic process). Niche theory assumes that species relative abundance (SRA) within a local community is driven by the performance-related traits of co-occurring species, therefore significant relationships between plant trait of co-occurring species and their corresponding SRA are expected^4–6^. Stochastic process asserts that all individuals are assumed to be functionally equivalent, therefore no significant plant trait – SRA relationship is expected^7,8^. Based on plant functional traits, it is easy to infer community assembly processes based on niche theory. Multi-trait functional diversity (FD) indices, including functional richness (FRic), functional divergence (FDiv), functional evenness (FEve) and so on, have been proved to be useful in revealing species coexistence processes and assembly rules^2,9^. A restricted range of trait values of co-occurring species, such as low FRic, is thought of as evidence of environmental filtering^3^ and an over-dispersion of trait values, take a high FDiv as an example, is considered as evidence of limiting similarity among co-occurring species^10^. And, the shifts in the FD could explain the consequences of environmental change on community assembly^11,12^. Community-weighted mean (CWM) trait value could help describe the central tendencies of a functional trait in a certain community^13^.

The community ecologist have found that the secondary succession could either converge or diverge with time relative to each other depending on a variety of factors including characteristics of the disturbance that initiates succession^14^. Traditional monitoring vegetation composition and each species’ abundance (or SRA) over time is helpful to judge the restoration outcomes. Not only that, estimating the response of species relative abundance (RSRA) through time or across treatments may provide a useful method to explore the deep reason of restoration outcomes^4,15–17^. If the RSRAs through time are significantly different between the restoration community and the stable climax community for almost species, more time is need for the restoration community to reach the climax status^16^. Alternatively, if no climax community, monitoring the RSRAs of dominant native species and problematic species (invasive species, or the dominant species in the degradation community) will always be an important component of evaluating restoration outcomes^18–20^. If the RSRA through time of the dominant native species is significantly higher than zero while that of the problematic species is significantly lower than zero, the community is restoring but does not reach the climax status on the condition that these findings were not influenced by year-to-year fluctuation^16^.

Large areas of terrestrial habitats are degrading due to global changes, especially in the arid and semi-arid regions. For example, in the semi-arid Inner Mongolia Steppe in the northern China, large areas of native climax communities, *S. grandis* – *L. chinensis* communities, degraded due to drought caused by climate change and over-grazing by cattle or/and sheep, with the loss of plant diversity and the decline of ecosystem functioning (e.g., net primary productivity). Consequently, these communities were replaced by other communities, such as *Cleistogenes squarrosa* communities^21^. In response, a number of restoration treatments, including enclosure no disturbance, mowing, reducing grazing intensity and so on, were carried out to restore the semi-arid grassland vegetation. In order to explore how different restoration treatments affect the community assembly processes and predict the restoration processes in the semi-arid grassland, communities treated by three different restoration treatments (enclosure, grazing and mowing treatments) were chosen in the distribution region of *S. grandis* – *L. chinensis* communities in the northern China. These communities were dominated by *C. squarrosa* before 2003, and restoration treatments have been implemented and extensive human activities have been forbidden in the study region since 2003. Every growth season from 2010 to 2012, we investigated plant traits, traits’ response, SRA and RSRA for each species in each community, estimated CWM and FD indices for each community. Comparing with the average precipitation (263.5 mm) of the last 30 years in the study region^22^, 267.9 mm in 2010, 226.7 mm in 2011, and 511.7 mm in 2012 represent normal precipitation, low precipitation and high precipitation, respectively. We explored the effects of treatment/year/species on these observed variables and tested the relationships between the trait values and the SRA, and between the trait responses and the RSRA across treatments/through time. Accordingly, these results would indicate which process(es), including stochastic process based on neutral theory, and environmental filtering and limiting similarity based on niche theory, played a more important role in affecting community assembly in the grassland restoration under each of the three different treatments.

## Results

### Responses of plant traits

In these communities, eleven species were common in 2010 and 2012, and only nine species were common in 2011 (Table 1). The plant height and the SLA were significantly (*P* < 0.01) affected by year (Y), treatment (T), species (S) and their interactions, and the one thousand grain weight was significantly (*P* < 0.05) affected by year, species and their interaction (Tables 1, 2).

**Table 1:** The species density, seed mass, plant height and specific leaf area of common species under three treatments during 2010 to 2012.

Species	FG	Seed diaspore type	One thousand grain weight (g)			Density (ramets/m^2^)									Plant height (cm)									Specific leaf area (SLA) (cm^2^/g)								
			2010	2011	2012	2010			2011			2012			2010			2011			2012			2010			2011			2012		
						En-	Gr-	Mo-	En-	Gr-	Mo-	En-	Gr-	Mo-	En-	Gr-	Mo-	En-	Gr-	Mo-	En-	Gr-	Mo-	En-	Gr-	Mo-	En-	Gr-	Mo-	En-	Gr-	Mo-
*Stipa grandis*	PG	Caryopsis	9.54	8.58	10.01	221	111	264	54	233	100	173	156	236	48.4	47.7	58.9	28.0	26.3	27.9	38.3	33.6	41.2	72.7	70.2	82.3	102.0	112.9	152.3	102.9	95.6	96.8
*Leymus chinensis*	PG	Caryopsis	2.04	1.94	2.10	89	36	23	452	199	83	282	236	145	29.4	23.4	26.5	32.4	21.0	22.3	36.4	29.5	39.9	77.0	89.4	73.8	95.5	111.2	184.8	116.9	138.5	106.6
*Agropyron cristatum*	PG	Caryopsis	2.19	2.23	2.10	161	116	220	118	70	198	68	106	185	24.1	26.1	25.5	29.4	30.4	19.3	29.7	26.5	35.1	96.0	78.0	74.8	74.2	104.3	155.8	134.2	138.3	134.1
*Cleistogenes squarrosa*	PG	Caryopsis	0.62	0.66	0.60	402	610	517	102	604	498	93	379	195	10.9	10.6	11.5	8.6	9.1	10.8	13.1	15.5	15.4	165.1	165.9	194.6	195.1	195.9	263.6	194.6	194.0	206.8
*Artemisia frigida*	PF	Achene	0.10	0.10	0.10	3	4	3	0	3	4	2	2	4	20.2	25.0	13.3		18.6	16.7	9.9	25.9	29.12									
*Serratula centauroides*	PF	Achene	7.68	7.23	7.92	3	4	1	4	3	3	3	3	4	36.0	30.2	43.6	26.0	12.1	12.8	44.2	31.1	41.7	89.7	88.9	76.6	100.7	102.2	155.1	120.0	114.5	108.7
*Carex korshinskyi*	PF	Caryopsis	2.15	1.94	2.26	30	12	18	68	25	35	109	66	36	9.8	8.7	9.3	11.0	11.7	8.5	19.6	18.0	18.8							128.0	133.7	129.6
*Allium tenuissimum*	PF	Seed	2.17	1.95	2.27	7	9	9	10	17	10	2	12	77	21.7	21.6	22.9	13.0	17.5	21.8	20.0	26.6	29.7	122.9	101.6	121.9	137.5	171.8	229.1	197.6	165.2	187.5
*Salsola collina*	A	Seed	0.15	0.14	0.16	43	80	20	0	3	1	11	38	12	22.1	22.5	19.9		9.5	7.0	12.8	23.7	27.4									
*Chenopodium glaucum*	A	Seed	0.47		0.49	20	14	13				254	21	116	16.5	15.3	14.6				29.6	18.0	34.2	162.0	130.4	165.2				141.2	172.4	143.6
*Chenopodium strictum*	A	Seed	0.21		0.22	2	5	2				59	7	28	7.8	11.8	16.7				33.6	17.5	33.8	158.2	159.5	216.8				173.1	208.4	194.1

(FG: functional group; PG: perennial graminoids; PF: perennial forbs; A: annuals plants).

**Table 2:** Summary of *F*-value and significance of different factors on the values and the response of species relative abundance, plant height, specific leaf area (SLA) and one thousand grain weight by general linear model.

Factor	Year (Y)	Treatment (T)	Species (S)	Y × T	Y × S	T × S	Y × T × S
**Species level**							
Species relative abundance (SRA)	2.364	0.834	95.55^***^	0.788	16.077^***^	15.365^***^	5.359^***^
Plant height	59.634^***^	6.187^***^	73.505^***^	4.726^**^	11.331^***^	2.7^***^	1.854^**^
Special leaf area (SLA)	151.22^***^	72.156^***^	190.4^***^	55.778^***^	10.273^***^	3.022^***^	3.01^***^
One thousand grain weight	3.448^*^	—	83.174^***^	—	6.459^***^	—	—
**Across treatments**							
Ln RR of SRA (RSRA)	2.747	0.015	15.348^***^	1.412	6.158^***^	2.112^*^	2.500^**^
Ln RR of plant height	2.074	6.168^*^	2.561^*^	2.663	3.293^***^	1.004	1.439
Plasticity of plant height	1.943	5.462^*^	2.397^*^	1.836	3.275^***^	0.827	1.702
Ln RR of SLA	69.415^***^	32.359^***^	3.477^**^	31.668^***^	6.399^***^	1.751	2.266^*^
Plasticity of SLA	79.796^***^	44.128^***^	4.395^***^	41.379^***^	7.266^***^	1.498	2.609^**^
**Through time**							
Ln RR of SRA (RSRA)	0.477	7.971^***^	22.627^***^	1.6	1.591	5.484^***^	3.615^***^
Ln RR of plant height	50.278^***^	1.797	13.06^***^	1.653	2.441^*^	3.584^***^	0.621
Plasticity of plant height	14.162^***^	3.149	10.622^***^	0.682	0.501	5.25^***^	0.307
Ln RR of SLA	0.127	27.766^***^	14.651^***^	37.076^***^	7.875^***^	6.188^***^	1.24
Plasticity of SLA	0.394	23.109^***^	8.885^***^	25.155^***^	3.896^**^	3.5^***^	1.136

*, ** and *** indicate the significant effect on variables at the 0.05, 0.01 and 0.001 level, respectively.

Both the Ln RR (the natural-log-transformed response ratio) and the plasticity of plant height across treatments were significantly (*P* < 0.05) affected by species and Y × S interaction. Both the Ln RR and the plasticity of plant height through time were significantly (*P* < 0.05) affected by year, species and T × S interaction, and the Ln RR of plant height through time was also significantly (*P* < 0.05) affected by Y × S interaction. Both the Ln RR and the plasticity of SLA across treatments were significantly (*P* < 0.05) affected by all these factors (year, treatment, species and their interactions) except T × S interaction. Both the Ln RR and the plasticity of SLA through time were significantly (*P* < 0.01) affected by all these factors (year, treatment, species and their interactions) except the factor of year and Y × T × S interaction (Tables 1, 2).

**Figure 1:**
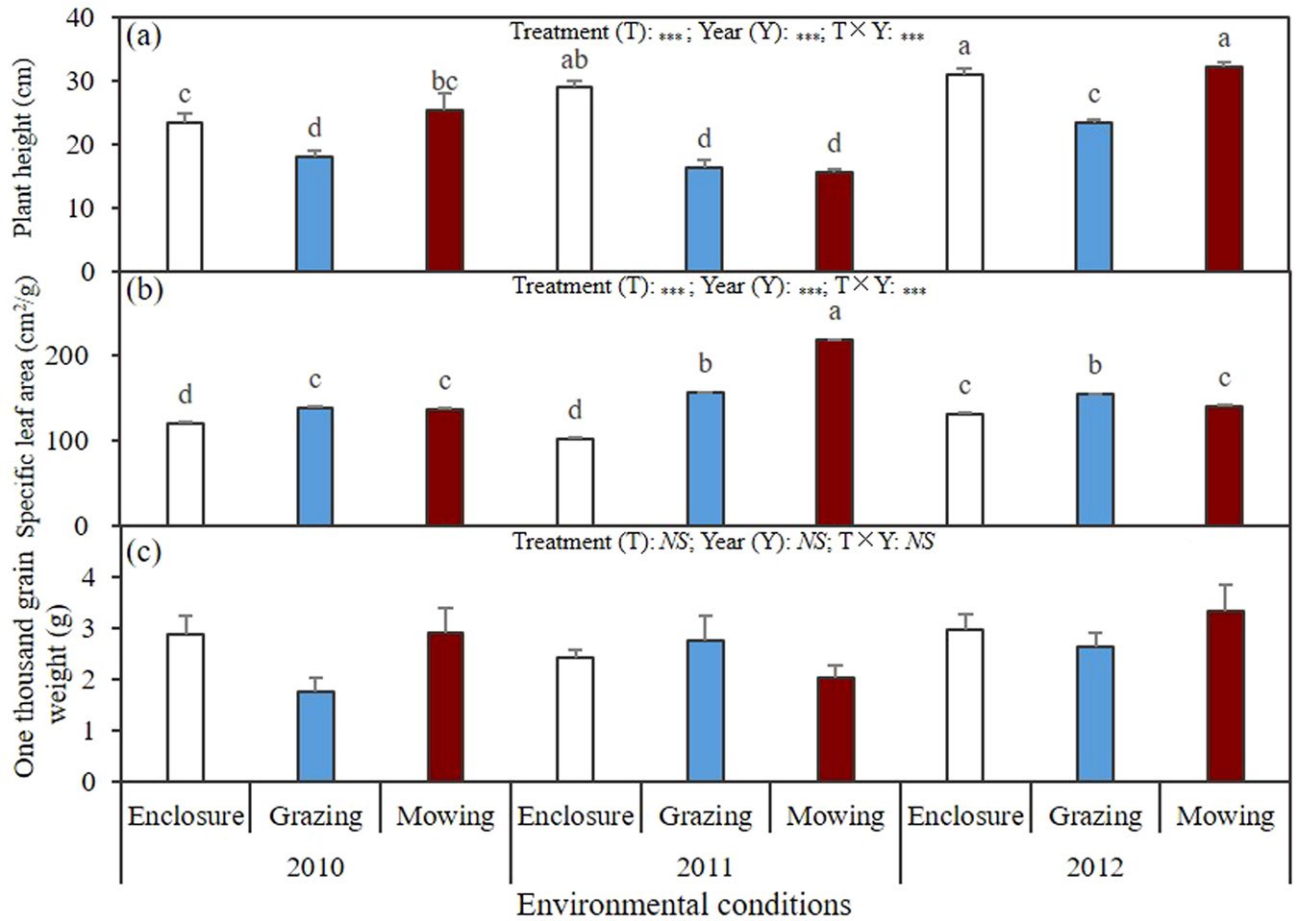
Results of analysis of variance on the community-weighted mean (CWM) of plant height (**a**), specific leaf area, SLA (**b**) and one thousand grain weight (**c**) in this study. NS indicates non-significant effect on the variable at the 0.05 level, and *, ** and *** indicate the significant effect on variables at the 0.05, 0.01 and 0.001 level, respectively. The values of Mean ± SE within each variable followed by the same letter are not significantly different at 0.05 level.

The CWM of plant height was significantly (*P* < 0.001) affected by treatment, year and their interaction. It was significantly (*P* < 0.05) lower within the grazing community than that within any of the other two communities in 2010 and 2012, and was significantly (*P* < 0.05) higher within the enclosure community than that within any of the other two communities in 2011 (Fig. 1a). The CWM of SLA was significantly (*P* < 0.001) affected by treatment, year and their interaction, and it was significantly (*P* < 0.05) lower within the enclosure community than that within any of the other two communities in any observation year. Compared with the CWM of SLA within the mowing community in the same year, the performance within the grazing community was significantly (*P* < 0.05) lower in 2011 and significantly higher in 2012 (Fig. 1b). The effects of treatment, year and their interaction on the CWM of one thousand grain weight was not significant (*P* > 0.05) (Fig. 1c).

### Response of species relative abundance (RSRA)

The SRA and the RSRA across treatments were significantly (*P* < 0.05) affected by species, Y × S, T × S and Y × T × S interactions (Table 2). There were four species, *S. grandis*, *C. squarrosa*, *Allium tenuissimum* and *Salsola collina*, whose RSRA to grazing (grazing vs enclosure) was significantly positive in at least one observation year or no difference with zero. There were three species, *L. chinensis*, *Carex korshinskyis* and *Chenopodium glaucum*, whose RSRA to grazing was significantly negative in at least one observation year or no difference with zero. The RSRA of *Artemisia frigida* to grazing was significantly positive in 2011, significantly negative in 2010, and no difference with zero in 2012. The significantly positive RSRAs to mowing (mowing vs enclosure) were shown by *Agropyron cristatum*, *C. squarrosa*, and *A. frigida* in 2011 and 2012, and *Allium tenuissimum* in 2011. The significantly negative RSRAs to mowing were shown by *L. chinensis* in any observation year, *Carex korshinskyi*, *Salsola collina* and *Ch. glaucum* in 2010 (Table 3).

**Table 3:** The results of the response of species relative abundance (RSRA) across treatments or through time calculated by one sample t-test with zero as test value. The ↑, ↓ and NS in the table indicate a significantly positive response, a significantly negative response and a non-significant response at the 0.05 level, respectively.

	RSRA to grazing (Grazing vs enclosure)			RSRA to mowing (Mowing vs enclosure)			RSRA to low precipitation (2011 vs 2010)			RSRA to high precipitation (2012 vs 2010)		
	2010	2011	2012	2010	2011	2012	En-	Gr-	Mo-	En-	Gr-	Mo-
*Stipa grandis*	NS	**↑**	NS	NS	NS	NS	**↓**	**↑**	NS	NS	NS	NS
*Leymus chinensis*	**↓**	**↓**	**↓**	**↓**	**↓**	**↓**	**↑**	**↑**	NS	**↑**	**↑**	**↑**
*Agropyron cristatum*	NS	NS	NS	NS	**↑**	**↑**	NS	NS	NS	NS	NS	NS
*Cleistogenes squarrosa*	**↑**	**↑**	**↑**	NS	**↑**	**↑**	**↓**	NS	NS	**↓**	**↓**	**↓**
*Artemisia frigida*	**↓**	**↑**	NS	NS	**↑**	**↑**	NS	NS	NS	NS	NS	NS
*Serratula centauroides*	NS	NS	NS	NS	NS	NS	NS	NS	NS	NS	NS	NS
*Carex korshinskyi*	**↓**	NS	**↓**	**↓**	NS	NS	NS	NS	NS	NS	NS	NS
*Allium tenuissimum*	NS	**↑**	**↑**	NS	**↑**	NS	NS	NS	NS	NS	NS	NS
*Salsola collina*	**↑**	NS	**↑**	**↓**	NS	NS	NS	NS	NS	NS	NS	NS
*Chenopodium glaucum*	NS		**↓**	**↓**	NS	NS	**↓**	**↓**	**↓**	**↑**	NS	**↑**
*Chenopodium strictum*	NS		NS	NS	NS	NS	**↓**	**↓**	**↓**	**↑**	NS	NS

The RSRA through time was significantly (*P* < 0.001) affected by treatment, species, T × S and Y × T × S interactions. The significantly (*P* < 0.05) positive RSRAs to low precipitation (2011 vs 2010) were shown by *S. grandis* under the grazing treatment and *L. chinensis* under the enclosure or grazing treatment. The significantly (*P* < 0.05) negative RSRAs to low precipitation were shown by *S. grandis* and *C. squarrosa* under the enclosure treatment, *Ch. glaucum* and *Ch. strictum* under any of these treatments (Table 3). The significantly (*P* < 0.05) positive RSRAs to high precipitation (2012 vs 2010) was shown by *L. chinensis* under any of these treatments, *Ch. glaucum* under the enclosure or mowing treatment, and *Ch. strictum* under the enclosure treatment. The RSRAs of *C. squarrosa* to high precipitation was significantly (*P* < 0.05) negative under any of these treatments (Table 3).

### Relationship between plant trait and SRA

There were significantly (*P* < 0.05) positive relationships between the plant height and the SRA in the enclosure community in 2011 and 2012. The relationships between the SLA and the SRA were significantly (*P* < 0.05) positive in the grazing community in 2010 and 2011 and in the mowing community in 2011, and significantly (*P* < 0.05) negative in the enclosure community in 2012. No significant relationship was found between the one thousand grain weight and the SRA in this study (Table 4).

**Table 4:** Pearson correlations between values of plant traits and their species relative abundance (SRA) under each treatment in each observation year.

Year	Treatment	Plant height	Specific leaf area (SLA)	One thousand grain weight
2010	Enclosure	0.056	0.075	0.096
	Grazing	−0.196	0.472*	−0.111
	Mowing	0.111	0.158	0.139
2011	Enclosure	0.475*	−0.219	−0.343
	Grazing	−0.122	0.607**	−0.029
	Mowing	−0.087	0.602**	−0.17
2012	Enclosure	0.388*	−0.458*	0.067
	Grazing	−0.117	0.079	0.022
	Mowing	0.11	−0.126	0.304

*, ** indicate significant relationships at the 0.05, 0.01 level, respectively.

### Relationship between response of plant trait and RSRA

The Ln RR of plant height was significantly (*P* < 0.05) positively correlated with the RSRA to grazing in 2010 and 2012, with the RSRA to mowing in 2011 and 2012, with the RSRA to low precipitation under the enclosure treatment, and with the RSRA to high precipitation under any of these treatments. The plasticity of plant height was significantly (*P* < 0.05) positively correlated with the RSRA to grazing in 2012, with the RSRA to mowing in 2011, and with the RSRA to high precipitation under any of these treatments. Both the Ln RR and the plasticity of SLA were significantly (*P* < 0.05) negatively correlated with the RSRA to grazing in 2011, with the RSRA to mowing in 2011, and with the RSRA to high precipitation under the enclosure treatment (Table 5).

**Table 5:** Pearson correlations between responses of plant height or specific leaf area and the corresponding RSRAs across treatments or through time.

		Ln RR of plant height	Plasticity of plant height	Ln RR of specific leaf area (SLA)	Plasticity of SLA
Response to grazing (Grazing vs enclosure)	2010	0.275^*^	0.240	−0.099	−0.112
	2011	0.278	0.298	−0.395^*^	−0.410^*^
	2012	0.591^***^	0.479^***^	−0.230	−0.218
Response to mowing (Mowing vs enclosure)	2010	0.166	0.145	−0.101	−0.096
	2011	0.381^*^	0.371^**^	−0.388^*^	−0.359^*^
	2012	0.258^*^	0.200	−0.037	−0.053
Response to low precipitation (2011 vs 2010)	Enclosure	0.406^*^	0.342	−0.108	−0.129
	Grazing	0.002	−0.144	0.168	0.181
	Mowing	0.156	0.134	0.240	0.230
Response to high precipitation (2012 vs 2010)	Enclosure	0.549^**^	0.559^***^	−0.341^*^	−0.308^*^
	Grazing	0.410^***^	0.394^***^	0.094	0.055
	Mowing	0.331^**^	0.300^**^	−0.167	−0.125

*, ** and *** indicate significant relationships at the 0.05, 0.01 and 0.001 level, respectively.

### Community functional diversity

**Figure 2:**
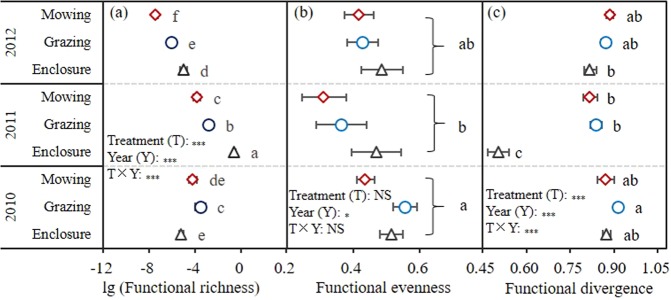
Results of analysis of variance on functional richness, FRic (**a**), functional evenness, FEve (**b**) and functional divergence, FDiv (**c**). NS indicates non-significant effect on the variable at the 0.05 level, and *, ** and *** indicate the significant effect on variable at the 0.05, 0.01 and 0.001 level, respectively. The values of Mean ± SE within each variable followed by the same letter are not significantly different at 0.05 level.

The functional richness and functional divergence were significantly (*P* < 0.05) affected by treatment, year and their interaction. The functional richness was significantly (*P* < 0.05) higher in 2011 than that in 2012, and the highest functional richness and the lowest functional divergence were found within the enclosure community in 2011. Only the factor of year significantly (*P* < 0.05) affected the functional evenness, and the lowest functional evenness was found in 2011 (Fig. 2).

## Discussion

The present study found that the percentage of significant plant trait - SRA relationships was higher than random expectation (5%), indicating that niche theory played more important role in affecting the community restoration in the study region. The environmental adaptability or tolerance of these co-occurring species could provide evidence that not all individuals were functionally equivalent and support the niche theory. The RSRAs across treatments were significantly different among the co-occurring species, implying the difference of adaptability and tolerance to disturbance among these species, which could cause different restoration outcomes due to the change of competition superiority of co-occurring species^17,23^.

In the enclosure community, the relative abundance of *S. grandis* or *L. chinensis* was significantly higher than that (<5%) before 2003, and the relative abundances of *S. grandis* and *L. chinensis* were the first two highest among all these species in 2012 (Table 1), implying the dominance of *S. grandis* and *L. chinensis* in this community. That is to say, the composition and structure in the enclosure community was close to the climax community in this region. The CMW of SLA was lower within the enclosure community than that within any of the other communities, and the SLA of the dominant native species, *S. grandis* and *L. chinensis*, was relatively lower than that of any of the other species, implying that the relatively lower SLA has the higher functional adaptability in this region. In semi-arid region, species with low SLA and therefore long-lived leaves can accumulate a greater mass of leaf, and long mean residence time of nutrients, made possible by leaf longevity, permits a progressively larger share of nitrogen pools to be sequestered^24^.

However, in the grazing/mowing community, the abundance of *C. squarrosa* was highest (>30%) among all these species (except in the mowing community in 2012), indicating that *C. squarrosa* but not *S. grandis* or *L. chinensis* was the dominant species. More time was needed for the grazing or mowing community to reach the status of *S. grandis* - *L. chinensis* community. The significant differences of RSRAs through time with zero suggested that these communities were more dynamic, or hysteresis may be occurring in response to the grazing/mowing disturbance^25,26^. The RSRAs of *L. chinensis* to grazing or mowing were significantly lower than zero while the RSRAs of *C. squarrosa* to grazing or mowing were significantly higher than zero except one that was no difference with zero, implying the relatively higher tolerance of *C. squarrosa* to grazing/mowing than *L. chinensis*. It has been reported that *C. squarrosa* showed higher tolerance to environmental disturbance or stress than *S. grandis* in the Inner Mongolia Steppe^27^. As a result, the relatively lower recovery (competitiveness) of *L. chinensis* population and the relatively higher competitiveness of *C. squarrosa* population in the grazing or mowing community than in the enclosure community. A disturbance could damage or destroy some vegetations, reducing the uptake of light, water and nutrient. Once resource uptake goes down for a time in an ecosystem, there will be more resource available and the competition among co-occurring species will decrease, thus the community is particularly vulnerable so that invasion or the dominant species replaced by high resource-capture species happens very easily^28^. Contrary to species with low SLA, species with high SLA, take *C. squarrosa* as an example, can capture resource rapidly, in sequence, become a superiority to occupy the gaps in a disturbance community such as a grazing or mowing community. Consequently, the disturbance could lead to increasing the competition superiority of *C. squarrosa*, and then increasing its SRA. In fact, when there is no disturbance, *C. squarrosa* is an inferior competitor; and even if disturbance exists, *C. squarrosa* is still an inferior competitor in high precipitation year^29^, as the results in Table 3 showed in this study. The significantly different of characteristic (adaptability, tolerance, SLA, plant height and so on) between *C. squarrosa* and *S. grandis* or *L. chinensis* could explain not only the degradation processes in this study region but also the restoration processes under different treatments.

Even though niche theory affected the community assembly in this region, the limiting similarity based on niche theory was more important in structuring the enclosure community and the environmental filtering based on niche theory played a more important role in driving the grazing or mowing community^3,10,30^. The FRic within the grazing/mowing community was lower than that within the enclosure community or that reported by other researchers^31,32^, therefore, it could be viewed as evidence of environmental filtering affecting the grazing/mowing community^3^. While the relatively higher FDiv within the enclosure community could be considered as evidence of limiting similarity among co-occurring species^10^. Moreover, the significantly positive plant height - SRA relationships in the enclosure community demonstrated that the higher species had more advantage, supporting that light competition was the driving force as community height and canopy density increased over years of enclosure and that limiting similarity and niche differentiation by light competition affected the community structure and processes in the enclosure community^33^. However, the dramatic inter-annual changes of the correlation coefficient or significance under the grazing/mowing treatment emphasized the importance of environmental filtering by inter-annual climatic changes in driving the community assembly^34^. For example, in the mowing community, the correlation coefficient between the SLA and the SRA was 0.602 and more higher than zero in 2011 but −0.126 in 2012 (Table 4), and similar results have been reported by many researchers^4,35^. From the restoration outcomes under different treatments, we conjectured that the limiting similarity played more important role in community closer to the climax community in the study region.

The present findings indicated that the factor of year was a significant factor interacting with restoration treatments in affecting the community dynamics in the semi-arid grassland. However, only the factor of year significantly affected the FEve, and the lowest FEve was found in 2011. According to viewpoint of Pakeman^11^, low level of FEve can be indicative of sites where the limiting similarity may be important in structuring the communities. Our finding implied that the relatively lower precipitation in 2011 enhanced the effect of limiting similarity on community assembly. Seed mass is quite a good indicator of a cotyledon-stage seedling’s ability to survive various hazards, representing a species’ chance of successful dispersing a seed into an establishment opportunity^36^. The relationship between one thousand grain weight and SRA was negative in 2011 but positive in 2012, suggesting the trend of community succession and the important effect of year-to-year (climatic) fluctuation on recruitment.

Our results supported that the restoration succession of the semi-arid grassland in northern China was sensitive to environmental conditions, including restoration treatments or year-to-year fluctuation, and demonstrated that long-term consecutive observations of plant traits and estimating FD indices are vital for understanding community assembly mechanisms. RSRAs across treatments or through time are helpful to calculate trajectories of community process and judge community state. Our findings provided a more comprehensive understanding of why some species are more abundant in one particular habitat but less abundant in another habitat, but also demonstrated the complexity of community assembly mechanisms and processes of grassland restoration in the northern China.

## Methods

### Study region

The study region (44°10′~44°20′N, 116°20′~116°30′E, 1110~1154 m a.s.l.) located at Xilingol League, the middle of Inner Mongolia Steppe of China where *S. grandis* – *L. chinensis* community is the typical and climax vegetation type. Before 2003, *S. grandis* – *L. chinensis* community in this region degraded heavily due to overgrazing by cattle or/and sheep, the relative abundance of *C. squarrosa* was more than 30% while that of *S. grandis* or *L. chinensis* was less than 5%. That is to say, *C. squarrosa* community replaced *S. grandis – L. chinensis* community. The soil type is typical chestnut soil (Chinese Soil Taxonomy System) and Calcic luvisol (FAO-UNESCO). The mean annual temperature is 0.8 °C, ranging from −39.9 °C in January to 37.4 °C in July, with approximately 106 frost-free days per year. Mean annual precipitation measured over the last 30 years is 263.5 mm^22^, falling mainly from July to September.

### Experimental design

In 2003, over-grazing was forbidden and three different restoration treatments were carried out in the study region. One-third was grazing plot, where light grazing was performed with approximately 3 sheep/ha from May to October. Two-thirds area was enclosed, and separated into enclosure plot and mowing plot. In the enclosure plot, there was no human-being disturbance; and in the mowing plot, the aboveground plants was mowed once in every autumn, leaving stubble height about 3~5 cm. In June 2010, eight 40 m × 200 m sampling belt were set up with 100-m interval, with each sampling belt covering the area of enclosure, mowing and grazing plot.

### Data collection

From 2010 to 2012, a sampling plot (5 m × 8 m) was set up per treatment per sampling belt at the end of July, avoiding any overlap with the sampling plots set up before. Four 1 m × 1 m quadrats per sampling plot were randomly arranged with the constraint that it was at least 0.5 m from the edge to avoid marginal effect, totalling 288 sampling quadrats during these three years.

The species were not present at all quadrats, species that appeared in higher than 10% sampling plots were thought of as common species and the functional traits of common species were collected for further analysis. These species presented more than 80% of the total above-ground biomass and vegetation cover, therefore, according to the viewpoint of Cornelissen *et al*.^37^, the result may provide useful information to scale-up the trait values from species to the community level.

A database of functional traits for each species observed during the plant composition surveys using standard methods^38^. We measured two traits from plants in the sampling plot (plant height and SLA), one trait in the study region (seed mass), and two categorical traits from literature (functional groups [perennial graminoids, perennial forbs and annual plants] and seed diaspore type [seed, caryopsis and achene]). We selected these traits because they are main predictors for RSRA across treatments or through time^2,36^. The plant species density and the plant height of 3~5 mature individuals per species were recorded within each quadrat. Mature, fully expanded leaves of each species were collected to measure SLA which was calculated as the ratio of leaf area to dry leaf mass^37^, three replicates per sampling plot. The leaves were scanned for measuring leaf area before being dried at 80 °C for 48 h and weighed for biomass to the nearest 10^−4^ g. For species with needle-like leaves or small leaves, four to six full leaves were thought of as one sample. The SLA values of both *A. frigida* and *Carex korshinskyi* showed a high random error, therefore, they were not used for further analysis. Fresh diaspores (referring to the seed and dispersal structures) were collected from May to September in 2010–2012. Diaspores were collected from as many individuals as possible (n > 20) in the study region. Structures having the function of contributing to dispersal were not included as part of seed mass^39^. One thousand grain weight was measured for each species as the characteristic of seed mass, ten replicates per year. We did not measure the one thousand grain weight by treatment, because there were not enough diaspores within the grazing community.

### Data analysis

Based on data collected from the same sampling plot, the mean values of plant height and SLA were calculated for each species, and the SRA of each species was calculated as the ratio of a given species’ density to the total species’ density. The CWM trait value was calculated with equation, $CW{M}_{j}=\mathop{\sum }\limits_{i=1}^{n}SR{A}_{ij}{T}_{ij}$, where *SRA*_*ij*_ is the SRA of the species *i* in sampling plot *j*, *T*_*ij*_ is the mean trait value of the species *i* in sampling plot *j*, and *CWM*_*j*_ is the community-weighted trait of sampling plot *j*. The Multi-trait FD indices, including FRic, FEve and FDiv, were calculated based on the species abundance and the ‘Gower’ distance matrices of the five traits (species functional group, diaspore type, plant height, SLA and one thousand grain weight). FRic represents the amount of functional space occupied by a species assemblage. FEve corresponds to how regularly species abundances are distributed in the functional space. FDiv defines how far high species abundances are from the center of the functional space. These three facets of FD are complementary and, taken together, describe the distribution of species and their abundances within the functional space^30^. If a plant trait is absent from one sampling plot, it is treated as missing data. If a species is absent from one sampling plot, it had a value of zero. These indices were calculated by using the ‘FD’ package^40^ in R.

The RSRA across treatments (grazing vs. enclosure treatment, mowing vs. enclosure treatment) or through time (2011 vs. 2010, 2012 vs. 2010) for each species was calculated by natural-log-transformed response ratio (Ln RR)^4^: *Ln RR* = *Ln [SRA*_*(G or M)*_/*SRA*_*E*_] or *Ln [SRA*_*(L or H)*_/*SRA*_*N*_]. *SRA*_*G*_, *SRA*_*M*_ and *SRA*_*E*_ were SRA of species *i* calculated in the same sampling belt in the same year in the grazing, mowing and enclosure plots, respectively; and *SRA*_*L*_, *SRA*_*H*_ and *SRA*_*N*_ were SRA of species *i* calculated in the same sampling belt under the same treatment in 2011, 2012 and 2010, respectively. Comparing with the average precipitation (263.5 mm) of the last 30 years^22^, 267.9 mm in 2010, 226.7 mm in 2011 and 511.7 mm in 2012 reflect the same, relatively lower and relatively higher precipitation, respectively. Therefore, the RSRA of 2011 vs 2010 and that of 2012 vs 2010 could be considered as the RSRA to low precipitation and to high precipitation, respectively.

The responses of plant height or SLA across treatments or through time were calculated for each species by two indices, LnRR (=*Ln [T*_*(G or M)*_/*T*_*E*_] or *Ln [T*_*(L or H)*_/*T*_*N*_])^4^ and trait plasticity (=[*T*_(*G* or *M*)_ − *T*_*E*_]/*T*_*E*_ or [*T*_(*D* or *W*)_ − *T*_*E*_]/*T*_*E*_)^41^. *T*_*G*_, *T*_*M*_ and *T*_*E*_ were the performance of plant height or SLA of species *i* measured in the same sampling belt in the same year in the grazing, mowing and enclosure plots, respectively; and *T*_*L*_, *T*_*H*_ and *T*_*N*_ were the performance of plant height or SLA of species *i* calculated in the same sampling belt under the same treatment in 2011, 2012 and 2010, respectively.

The RSRAs across treatments or through time obtained for each species are symmetric around zero. The difference significance with 0 for each species’ RSRA was examined by *t*-test, where a significantly higher value than zero, a significantly lower value than zero and no difference with 0 indicate a positive response, a negative response and a non-significant response across treatments or through time, respectively. In addition, the effects of treatment, year, species and their interactions on plant traits, traits’ responses, SRA and RSRA were analyzed by linear mixed model restricted maximum likelihood (REML) with species as a fixed factor, treatment and year as random factors. The REML analyses allow the specification of fixed and random model structures for the analysis of unbalanced data set. Four variables, including SRA, plant height, SLA and one thousand grain weight, were log-transformed to meet the normality. Moreover, the effects of year, treatment and their interaction on the three FD indices (FRic, FEve and FDiv) and the CWM of each trait were analyzed by general linear model - univariate analysis with treatment and year as fixed factors and the FRic being log-transformed. At last, the relationships between each plant trait (plant height, SLA and one thousand grain weight) and the SRA, and between responses of plant height or SLA and RSRA were analyzed by Spearman correlation analysis. All these analyses were performed by using SPSS 21.0 (IBM, Chicago, IL, USA).
